# Ankylosing Spondylitis in Iran; Late Diagnosis and Its Causes

**DOI:** 10.5812/ircmj.11798

**Published:** 2014-04-05

**Authors:** Mehrzad Hajialilo, Amir Ghorbanihaghjo, Alireza Khabbazi, Suosan Kolahi, Nadereh Rashtchizadeh

**Affiliations:** 1 Connective Tissue Diseases Research Center, Tabriz University of Medical Sciences, Tabriz, IR Iran; 2 Biotechnology Research Center, Tabriz University of Medical Sciences, Tabriz, IR Iran; 3 Drug Applied Research Center, Tabriz University of Medical Sciences, Tabriz, IR Iran

**Keywords:** Spondylitis, Ankylosing, Delayed Diagnosis, Back Pain

## Abstract

**Background:**

Ankylosing spondylitis (AS) is a chronic destructive and inflammatory disease of the axial skeleton manifested by back pain and progressive stiffness of the spine.

**Objectives:**

The aim of the present cross-sectional study was to evaluate and identify factors leading to delayed diagnosis of AS in Iranian patients.

**Patients and Methods:**

Sixty patients, (53 males, 7 females) with a diagnosis of AS according to the modified New York criteria were recruited. Diagnosis delay was defined as the interval between a patient’s first spondyloarthritic symptoms [inflammatory back pain (IBP), inflammatory arthritis, enthesopathy and uveitis] and a correct diagnosis of AS.

**Results:**

The average age of patients at diagnosis of AS was 36.4 ± 4.5 years and the average of delay in diagnosis was 6.2 ± 3.5 years. The most common diagnosis at the first visit was disc herniation (68.3%). Delay in diagnosis of Human Leukocyte Antigen (HLA-B27) positive and negative patients were 4.6 ± 2.2 years and 10.1 ± 3.2 years, respectively (P = 0.0001). Diagnosis delay in patients with morning stiffness and IBP were significantly shorter than that of patients without these symptoms (P = 0.0001 and P = 0.001, respectively). Patients with uveitis had the shortest diagnosis delay (P = 0.02). The Bath Ankylosing spondylitis disease activity index (BASDAI) was not significantly different in early (< 3years) and late (> 3years) diagnosis (3.3 ± 0.9 and 3.6 ± 0.7, respectively) (P = 0.18), but the Both ankylosing spondylitis functional index (BASFI) was significantly different between them (3.3 ± 1.0 and 4.1 ± 0.7 respectively) (P = 0.001).

**Conclusions:**

In this study, delay in diagnosis was similar to other studies. Educating physicians to careful history taking especially in the case of IBP, non-musculoskeletal symptoms such as uveitis and precise physical examination are important in early diagnosis.

## 1. Background

Ankylosing spondylitis (AS) is characterized by inflammation and new bone formation in axial skeleton and enthesis. Peripheral joints and other organs such as skin, eye and heart may be involved. The prevalence of AS closely parallels the frequency of Human Leukocyte Antigen (HLA-B27) and varies from 0.1% to 1.2% in different populations (1). Some subtypes of HLA-B27 have stronger association with AS (2, 3). AS is more common in men with a male: female ratio of approximately 2:1 and typically seen during the third decade of life (4). The disease may also remain active over many years and life expectancy is somewhat reduced in particular after 10 years (5). Early diagnosis and treatment of AS is important because it can reduce the disease disability. There is no strong evidence to suggest that any of conventional disease -modifying antirheumatic drugs (DMARD) including sulfasalazine and methotrexate may alter or inhibit the inflammation seen in the spine. Given the efficacy of new drugs such as anti-tumor necrosis factor alpha agents (6, 7), early diagnosis of AS is very important. AS diagnosis at an early stage depends primarily on a careful history taking and physical examination. Diagnosis is delayed especially in primary and secondary care units (8, 9), since there are not unique and specific symptoms for the precise diagnosis of AS (10). A definite diagnosis of AS in the past years was based on the radiographic evidence of bilateral sacroiliitis (attention to New York criteria). The plain anteroposterior view of the pelvic is usually not a means to diagnose early sacroiliac disease (11). There is considerable-intra and inter observer variation in the radiographic diagnosis of sacroiliitis for both conventional pelvis films of the sacroiliac joints. It may take years before the inflammatory process leads to the appearance of radiological signs in the sacroiliac joints (12). In the context of all-inflammatory rheumatic diseases, there is an unacceptably long delay between the onset of symptoms and the time of diagnosis for AS; an average interval of 8–11 years has been reported (13).

## 2. Objectives

The aim of the present cross-sectional study was to evaluate and identify factors leading to delay in the diagnosis of AS in Iranian patients.

## 3. Patients and Methods

This was a cross sectional study performed in Emam Reza Hospital-Tabriz, Iran. The ethics committee of Tabriz University of Medical Sciences reviewed and approved the present study, which complies with the Declaration of Helsinki. Informed consent was obtained from all the participants in the study. Sixty patients (53 males, 7 females) were selected in university rheumatology clinics, Tabriz- Iran. The Modified New York criteria was used to diagnosis AS. All patients previously diagnosed as AS were rechecked and confirmed by two rheumatologists (14).

In this study we asked questions about many aspects of the disease including first manifestation of disease, first diagnosis, number of visits of patients by different physician before a correct diagnosis, duration of the disease, age at diagnosis time, presence and duration of morning stiffness, and presence of sacroiliac pain, low back pain, peripheral arthritis, uveitis and treatment. Diagnosis delay was defined as the interval between a patient’s first spondyloarthritic symptoms [Inflammatory back pain (IBP), inflammatory arthritis, enthesopathy and uveitis] and a correct diagnosis of AS. Criteria for inflammatory back pain were:

1. morning stiffness of > 30 minutes
2. improvement in back pain with exercise but not with rest
3. awakening because of back pain during the second half of the night only
4. and alternating buttock pain (15).

We assumed 6 mg/L as the cut of level for CRP based on the recommendation of manufacturer kits. Regarding patients' age and gender, we chose 30 mm/h for ESR level. Presence of AS (primary or secondary to inflammatory bowel disease, psoriasis or reactive arthritis) in the first or second-degree family of patients confirmed by rheumatologist was considered as positive family history. All patients were examined precisely by a rheumatologist. Disease activity was assessed by using the Bath ankylosing spondylitis disease activity index (BASDAI) (16), and function by the Bath ankylosing spondylitis functional index (BASFI) (17).

In all patients, radiography of sacroiliac joints and lumbar spine were taken. Peripheral joints radiography in the case of their involvement was also performed. Grading sacroiliitis, was according to the New York criteria (0: Normal, 1: Suspicious, 2: Minimal sacroiliitis, 3: Moderate sacroiliitis, 4: Ankylosis) All radiographies were reported by a radiologist. Erythrocyte sedimentation rate (ESR), serum C-reactive protein (CRP) and human leukocyte antigen B27 (HLA-B27) were determined. All data were entered the SPSS software package version 13 for Windows (SPSS Ins, Chicago, IL) for statistical analysis. Results were expressed as mean ± SD, numbers, and percent when appropriate. The Student’s t test was used for statistical analyses and P < 0.05 was considered significant

## 4. Results

Demographic characteristics of the patients are shown in Table 1. The male/female ratio was 0.13. The mean age at the time of correct diagnosis was 36.4 ± 4.5 years and diagnosis delay was 6.2 ± 3.5 years. Ninety percent of patients (n = 54) had conventional treatments like as Sulfasalazine, Methotrexate and NSAIDS (Nonsteroid anti-inflammatory drugs) and only 10% (n = 6) of them received TNF-a blockers including Etanercept and Infliximab. The common first symptoms at disease presentation were: low back pain (51.7%), buttock pain (23.3%), morning stiffness (20%) and peripheral arthritis (5%).

The average number of physicians who visited patients before the correct diagnosis was 4.1. Only four patients, one by a neurosurgeon and three by rheumatologists were correctly diagnosed at the first visit. The diagnoses at the first visit were disk herniation (68.3%), Ankylosing Spondylitis (6.6%), osteoarthritis (5%), sprain (5%), psychogenic pain (5%) and heel spur (3.3%). The correct diagnosis by Rheumatologists was higher than that of others (83.3%). The percentage of correct diagnosis was 5% by neurosurgeons and 11.7% by Internists, rehabilitationist, neurologists and general physicians. The average of diagnosis delay according to the manifestations of disease is shown in Table 2. The diagnosis delay was 4.8 ± 1.9 years and 8.7 ± 4.4 years in the presence or absent of inflammatory back pain respectively and the difference was statistically significant (P = 0.001). Peripheral arthritis was associated with a more diagnosis delay while morning stiffness was associated with a shorter delay of diagnosis (P = 0.0001).

In this study diagnosis delay was not sex dependent (P = 0.14). When patients had heel pain, diagnosis delay was very long (13 ± 0.0 years). The shortest (2.4 ± 0.3 years) delay was when patients had anterior uveitis. The average of diagnosis delay according to some laboratory data is shown in Table 3. Patients were divided into two groups of HLA-B27 positive (71.6%) and HLA-B27 negative (28.3%). The average of diagnosis delay was significantly different between HLA-B27 negative and HLA-B27 positive patients (P = 0.0001). Delay of diagnosis was shorter when ESR was above 30 mm/h and CRP above 6 mg/L in comparison when they were near the normal levels (P = 0.0001 and P = 0.036 respectively).

According to diagnosis delay we divided patients into two groups: early diagnosis < 3 years and late diagnosis > 3 years. BASDAI in early diagnosis was 3.3 ± 0.9 years and in late diagnosis was 3.6 ± 0.7 years and there were no significant differences between them (P = 0.18). On the other hand, BASFI were 3.3 ± 1.0 and 4.1 ± 0.7 on early and late diagnosis respectively with significant differences (P = 0.001). Radiographic findings in our patients were: Sacroiliitis (Grade: 2 = %33.5, Grade: 3= 38.65%, Grade: 4 = 27.82%), squaring (18.33), Romanus lesion (6.6%), Bamboo spine (6.6%) and enthesopathy (6.6%) (Table 4).

**Table 1.** Demographic Features and Biochemical Parameters in Studied Patients ^a, b^

Variable	Value
**Number of patients**	60
**Sex **	
Female	7 (11.7)
Male	53 (88.3)
**Mean age at the time of correct diagnosis, y**	36.4 ± 4.5
**Delay diagnosis, age between the onset of spondyloarthropathy**	6.2 ± 3.5
**Symptoms and correct diagnosis, y**	
HLA-B27 positive	43 (71.7)
HLA-B27 negative	17 (28.3)
**Conventional treatment (NSAID, Sulfasalazine Methotrexate)**	54 (90)
**TNF-α blockers**	6 (10)

^a^ Abbreviations: HLA-B27, Human Leukocyte Antigen; TNF-α, Tumor necrosis factor alpha.

^b^ Data are presented as No. (%) or mean ± SD.

**Table 2.** Comparison of the Average of Diagnosis Delay According to the Manifestation of Disease ^a, b^

Clinical Features	No. (%)	Average Diagnosis delay, y	P Value
**Inflammatory back pain**			0.001
Positive	39 (65)	4.8 ± 1.9	
Negative	21 (35)	8.7 ± 4.4	
**Buttock pain**			0.07
Positive	30 (50)	5.3 ± 3.8	
Negative	30 (50)	7.0 ± 3.1	
**Peripheral arthritis involvement**			0.0001
Positive	10 (16.7)	11.3 ± 1.8	
Negative	50 (83.3)	5.1 ± 2.8	
**Morning stiffness**			0.0001
Positive	43 (71.7)	4.6 ± 2.2	
Negative	17 (28.3)	10.1 ± 3.2	
**Anterior uveitis**			0.02
Positive	4 (6.7)	2.4 ± 0.3	
Negative	56 (93.3)	6.4 ± 3.5	
**Heel pain**			0.004
Positive	2 (3.3)	13.0 ± 0.0	
Negative	58 (96.7)	5.9 ± 3.4	
**Sex**			0.14
Female	7 (11.7)	8.0 ± 4.7	
Male	53 (88.3)	5.9 ± 3.3	
**Family history**			0.64
Positive	18 (30)	6.5 ± 3.4	
Negative	42 (70)	6.0 ± 3.6	

^a^ P <0.05 was considered significant.

^b^ Data are presented as No. (%) or mean ± SD.

**Table 3.** Comparison of the Average of diagnosis delay according to the HLA-B27, ESR and CRP ^a, b^

	Number of Patients	Average Diagnosis Delay, y	P Value
**HLA-B27**			0.0001
+	43	4.6 ± 2.2	
-	17	10.1 ± 3.2	
**ESR, mm/h**			0.0001
> 30	34	4.8 ± 2.7	
< 30	26	7.9 ± 3.8	
**CRP, mg/L**			0.036
> 6	45	5.6 ± 3.3	
< 6	15	7.8 ± 3.7	

^a^ Abbreviations: CRP, C-reactive protein; ESR, erythrocyte sedimentation rate; HLA-B27, human leukocyte antigen.

^b^ Data are presented as mean ± SD.

**Table 4.** Comparison of the BASDAI and BASFI With Types of Treatment (Conventional Versus TNF Blocker), Diagnosis Time (Early Versus Late) and HLA-B27 ^a, b^

Clinical feature	Number of Patients	BASDAI	BASFI
**Diagnoses, y**			
Early < 3	14	3.3 ± 0.9	3.3 ± 1.0
Late > 3	46	3.6 ± 0.7	4.1 ± 0.7
P value		0.18	0.001
**Treatment**			
Conventional	54	3.6 ± 0.7	4.1 ± 0.7
TNF blocker	6	2.7 ± 0.3	2.3 ± 0.6
P value		0.009	0.001
**HLA-B27**			
Negative	17	3.8 ± 0.9	4.1 ± 0.8
Positive	43	3.4 ± 0.6	3.9 ± 0.9
P value		0.06	0.37

^a^ Abbreviations: BASDAI, Bath ankylosing spondylitis disease activity index; BASFI, Bath ankylosing spondylitis functional index; HLA-B27, Human leukocyte antigen; TNF, tumor necrosis factor alpha blocker.

^b^ Data are presented as mean ± SD

## 5. Discussion

Ankylosing spondylitis (AS) is a chronic destructive and inflammatory disease of the axial skeleton manifested by back pain and progressive stiffness of the spine. Early diagnosis is important, because new drugs such as antitumor necrosis factor (TNF) agents are effective on reducing disease progression, enthesitis, spinal mobility and arthritis (18-20). For established disease, the classification criteria diagnostically useful are the modified New York criteria, which combine back pain with limitation of range of motion, and with radiological sacroiliitis (14). For the diagnosis of early disease, the most useful SpA classification criteria are the Assessment of Spondyloarthritis International Society (ASAS) classification criteria for axial spondyloarthritis (21).

AS diagnosis at an early stage depends primarily on a careful history taking and physical examination. Inflammatory back pain is an important clinical symptom of AS and there are no costs to evaluate this feature. Inflammatory back pain can be diagnosed with a sensitivity of 95% and specificity of 85 to 90% (13). Braun et al. emphasized on inflammatory back pain in primary care for early diagnosis (22). In this study, delay in diagnosis was similar to other studies (23, 24). We think that its cause is direct visit by rheumatologists without consulting their family physicians because we have not regular referral program in our health care system.

The first common symptom presented in our study was inflammatory lumbar pain (51.7%) and the common diagnosis at first visit was disc herniation (63%). Unfortunately, it emphasizes that physicians are not careful about IBP. Only 16.7% of our patients were correctly diagnosed by non-rheumatologist physicians. On the other hand we must know that 20-30% of AS patients may also present with mechanical back pain (13). In this study the shortest time to correct diagnosis based on symptoms, was anterior uveitis and physicians should be careful in case of extra spinal signs of AS. Our study showed that there is a long diagnosis delay in the presence of peripheral arthritis and heel pain (P = 0.0001, P = 0.004, respectively). We think that primary care physicians and non-rheumatologists do not consider AS in differential diagnosis when patients present with enthesopathy and arthritis. This is more prominent in peripheral arthritis because there are many etiologies for inflammatory arthritis. On the other hand, when patients have anterior uveitis the delay of diagnosis is shorter. Probably it is due to recommendation or consultation of ophthalmologist to investigate for spondyloarthropathies. 

In our study the average diagnosis delay was 4.6 ± 2.2 years and 10.1 + 3.2 years in HLA-B27 + and HLA-B27–patients respectively (P = 0.0001). HLA-B27 is not a criterion for the diagnosis of AS patients in the modified New York criteria, but used in the ASAS criteria. It seems that when physicians visit HLA-B27 positive patients, they mostly think of AS. In Feldtkeller et al. study, the average diagnosis delay were 11.4 and 8.5 years, in HLA-B27 negative and HLA-B27 positive patients respectively (25).

Sonkar study showed that frequency of HLA- B 27 positivity is high in SSA (Seronegative Spondyloarthropathies) in childhood and in young adult males. Sonkar et al. study showed that HLA- B27 positive patients have more severe disease and systemic manifestation (26). Our results showed no significant differences between BASDAI and BASFI based on HLA-B27 (P = 0.06 and P = 0.17 respectively).

A significant association was found between elevated ESR and shorter diagnosis delay. It may reflect that when physicians visit a patient with elevated ESR they are more sensitive to investigated inflammatory diseases such as AS. Based on early (< 3 years) or late (> 3 years) diagnosis delay, there were no significant differences in BASDAI score (P = 0.18), but in BASFI it was very significant (P = 0.001). Because many of our patients were diagnosed after several years, at the time of their disease was not very active based on BASDI but it was suspected functional status of patients decreased during disease progression. Ineffective treatment may be another cause of no differences in BASDI for early or late diagnosis. When we compared BASDAI and BASFI in the two groups (receiving Anti-TNF and or not) of patients, we found significant differences between them (P = 0.009, P = 0.001 respectively). AS patients with shorter duration of disease are more likely to respond to anti-TNF agents than those with long-standing disease (27).

In this study, all the patients at the diagnosis had sacroiliitis at radiography but we could not establish when radiographic changes occurred. In the modified New York criteria, sacroiliitis is essential for diagnosis. However, in many patients with AS, it may take years from the onset of AS symptoms to appear. It must be emphasized that if there was IBP in the early period of AS, absence of sacroiliitis should not be considered to rule out diagnosis (28). In patients suspected to AS without plain radiographic evidence, magnetic resonance imaging (MRI) would be helpful. MRI of the sacroiliac joints is not necessary in evaluating patients for possible AS if there is already evidence of sacroiliitis on plain radiographs or if the patient has already fulfilled the ASAS criteria by other parameters. A consensus opinion on how to diagnose sacroiliitis based on MRI has been issued by the Assessment of Spondyloarthritis International Society (ASAS)/Outcome Measures in Rheumatology (OMERACT) MRI working group (29).

Family history of spondyloarthritis is a key element in the evaluation of patients for AS. A family history of AS can be found in 15% to 20% of cases (30). In our study there was no significant difference in diagnosis delay between positive and negative family history (P = 0.64). It may be due to lack of attention of physicians in recording family history. One molecular feature identified as a Vitamin D3 metabolite-(23S, 25R)-25-hydroxyvitamin D3 26,23-peroxylactone-was down-regulated in AS. The ratio of this vitamin D metabolite versus vitamin D binding protein serum levels was also altered in AS as compared with controls. In the future using serum biomarkers may facilitate early AS diagnosis (31).

Educating physicians to take careful history especially about IBP, attention to non-musculoskeletal symptoms such as uveitis, arthritis, enthesopathy, precise physical examination and HLA-B27 are important in early and definite diagnosis of AS. Patients with arthritis and enthesopathy should be referred to rheumatologists since the patients had longest diagnosis delay. However, our findings are limited by the small number of patients included and we need to perform further studies with a greater number of patients (Table 4).
